# Analysis of radiation oncology integration within general practitioners’ daily patient care: a cross-sectional survey in Germany

**DOI:** 10.1007/s00066-024-02352-1

**Published:** 2025-01-16

**Authors:** Sophia M. Leiss, Helen X. Hou, Steffi Pigorsch, Kai Borm, Jan Peeken, Antonius Schneider, Stephanie Combs, Andreas Dinkel, Denise Bernhardt

**Affiliations:** 1 Department of Radiation Oncology, Klinikum rechts der Isar, School of Medicine and Health, Technical, University of Munich (TUM), Ismaninger Str. 22, 81675 Munich, Germany; 2 Department of Psychosomatic Medicine and Psychotherapy, Klinikum rechts der Isar, School of Medicine and Health, Technical University of Munich (TUM), Langer Str. 3, 81675 Munich, Germany; 3 Institute of General Practice and Health Services Research, School of Medicine and Health, Technical University of Munich (TUM), Orleansstr. 47, 81667 Munich, Germany; 4 Department of Radiation Sciences (DRS), Institute of Radiation Medicine (IRM), Ingolstädter Landstr. 1, Neuherberg, Germany; 5 Partner Site Munich, Deutsches Konsortium für Translationale Krebsforschung (DKTK), Munich, Germany

**Keywords:** Primary care integration, Interdisciplinary collaboration, Cancer patient management, Radiotherapy access, Healthcare survey

## Abstract

**Purpose:**

General practitioners (GPs) play a crucial role in providing interdisciplinary care for radiation oncology patients. This study aims to understand the specific needs and challenges faced by general practitioners in Germany when treating oncology patients.

**Methods:**

A comprehensive web-based questionnaire with 24 items was disseminated to GPs in Germany via email using *survio.com*. The survey collected data on demographics, qualifications, clinical experiences, decision-making involvement, and symptom recognition. It specifically examined post-radiotherapy care and the use of specialized palliative homecare networks (SAPV). Statistical analyses were descriptive. The survey was open from July 4 to August 9, 2023.

**Results:**

A notable majority of general practitioners displayed confidence in their understanding of cancer-related symptoms, with over half (54.6%) rating their knowledge with 4 out of 5. This level of self-assessed expertise extended to their capacity to address the needs of cancer patients (53.8%), although 67% express a need for further education in specifically radiotherapeutic side effects. Satisfaction with SAPV networks was high, and 72.3% of respondents were actively involved in palliative care, compared to only 45.6% in managing radiation therapy. Notable challenges included inadequate communication with specialists, insufficient staffing, and under-recognition of GPs’ roles in oncology care.

**Conclusion:**

The study highlights a paradox where GPs show high engagement in palliative care but limited involvement in radiation therapy management due to communication gaps and professional development needs. Addressing these disparities through targeted initiatives and fostering a collaborative care model is essential to amplify the important role of GPs, ensuring more integrated and effective patient care.

## Introduction

General practitioners (GPs) are often the initial point of contact for patients with symptoms [1]. They handle a range of tasks, spanning primary to secondary care management within the healthcare system and extending to the coordination of care with family caregivers [2, 3]. Oncologists tend to view their roles and those of GPs as distinct, with the former focusing on biomedicine and the latter on psychosocial support [4]. However, this division is often more nuanced in practice [5], and the complex support required by cancer patients compels GPs to extent their reach beyond conventional clinical interactions into realms of extensive follow-up care and education [6].

The German Cancer Society [7] notes that a significant majority of palliative care patients and survivors, especially in less urbanized settings, predominantly rely on GPs as their primary healthcare contact. The National Cancer Plan, initiated by the German Ministry of Health in 2008, was tasked with enhancing early cancer detection and care management. Despite lengthy deliberations, its sub-goal of “Cross-sectoral, integrated oncological care is guaranteed”—a mandate for improved networking and interdisciplinary collaboration—was relegated to other sub-goals and not directly pursued [8].

Effective GP–specialist communication is crucial in cancer management, especially in shared follow-up models involving radiation therapy (RT) [9]. In a previous study from 2021, it was advocated to introduce health technologies to facilitate bidirectional communication between GPs and radiation oncologists for the exchange of patient information. However, the precise operational dynamics of these technologies and guidelines demand rigorous empirical inquiry [10]. Despite RT’s efficacy, GPs’ referral practices and confidence in radiotherapy, especially in palliative care, are critical [11]. GP referrals are often hindered by a lack of detailed understanding about the intricacies of radiation therapy, including its potential risks and benefits. This knowledge gap poses a significant obstacle to fully leveraging RT in palliative care, necessitating targeted educational strategies to enhance GPs’ competency in this area [11, 12]. In this context, a German survey provides an important perspective on the intersection of competencies between radiation oncologists and palliative care. Findings indicate that those radiation oncologists with an additional qualification in palliative care were more adept at and willing to engage in interdisciplinary care. Furthermore, the successful integration of palliative care within radiation oncology is frequently challenged by practical constraints, including time limitations and organizational barriers [13]. Specialized palliative homecare networks (SAPV) are being provided at the patient’s home and multidisciplinary teams are involved. In contrast to general outpatient palliative care (AAPV)—where the GP is greatly involved in the patient’s care—SAPV specifically works with patients whose needs are more complex. While GPs play a key role in AAPV, they prescribe SAPV and remain in contact and coordinate multidisciplinary teams [12, 14].

We conducted a survey to investigate the needs and challenges of GPs in the oncologic care setting. The study aims to evaluate the status of healthcare services for cancer patients in GP practices, focusing on their experiences, perceived competencies, needs, and involvement, particularly in palliative care and radiation therapy.

## Methods

### Design and procedure

For this cross-sectional study, an online survey was distributed via the personal or office email of general practitioners in Germany on July 4, 2023. One reminder was sent out. On August 9, 2023, the survey was closed, and data analysis began.

The construction of the questionnaire was a collaborative effort involving the supervisor of this thesis, Prof. Dr. Andreas Dinkel (psycho-oncologist), and PD Dr. Denise Bernhardt (radio-oncologist), the study’s principal investigator. Additionally, the instrument underwent a review by Prof. Dr. Antonius Schneider, Director of the Institute of General Medicine at the TU Munich. This allowed a practical approach where experienced physicians from different fields contributed to the development of the survey. The questionnaire was distributed nationally via the online platform *survio.com*, utilizing an emailed invitation link. Contact emails were procured from *ArztData AG*, a database of German medical professionals. Due to the possibility of multiple contacts per practice, some physicians may have received more than one invitation. The study focused on German general practitioners working in the following settings: group practice (*Gemeinschaftspraxis*), ambulatory healthcare center (*Medizinisches Versorgungszentrum*, MVZ), joint practice (*Praxisgemeinschaft*), own office (*KV-Sitz*), and salaried positions in a practice.

Participants were informed about the survey’s anonymity and voluntary nature.

### Measure

A total of 24 questions were posed in this survey, employing a combination of single (*n* = 21) and multiple-choice (*n* = 3) formats. All questions were structured as closed ended, apart from the final inquiry. Furthermore, 19 items were designated as compulsory. Most of the questions (*n* = 21) were formatted to elicit a single-choice response. In addition, six questions allowed respondents to select “other” and elucidate their choice via a free-text option. The survey had five sections capturing GPs’ experiences and perspectives in oncologic care. The numbers in parentheses refer to the item numbers in the questionnaire (see supplemental material):

1. **Professional demographics ** (five items): initial questions gathered data on qualifications (1: Question 5), clinical tenure (1: Q4), workplace type (1: Q3), gender (1: Q2), and age (1: Q1).
2. **Clinical experiences and perspectives ** (five items): exploration of GPs’ familiarity with treating patients with cancer (4: Q6, Q7, Q8, Q9) and patient expectations (1: Q10).
3. **Decision-making and perceived competences and needs ** (nine items): knowledge of patient symptoms (1: Q11) and needs (1: Q12); follow-up care, specifically palliative care and radiotherapy (5: Q13, Q14, Q15, Q16, Q19); perceived needs for additional training in radiotherapeutic side effects (1: Q21); and assessment of confidence in decision-making with radiotherapeutic patients (1: Q20).
4. **Interprofessional communication** (four items): preferences in workload (2: Q17, Q18) and communication with oncologists (1: Q22) and primary responsibilities in oncologic care (1: Q23) were examined.
5. **Further comments ** (one item): an open-ended question concluded the survey (1: Q24).

### Statistical analysis

Data analysis was performed using IBM SPSS Statistics (version 29.0; IBM Corp., Armonk, NY, USA). The data were analyzed descriptively, providing frequency distributions.

Responses to the open-ended question were qualitatively analyzed. Categories were formed based on thematic similarity by the first author (SL).

This study was approved by the ethics committee of the Technical University of Munich, Germany (2023-99-S-S-KK). All examinations and evaluations were performed following institutional guidelines and the Declaration of Helsinki of 1975 in its most recent and updated version.

## Results

### Study participants

**Fig. 1:**
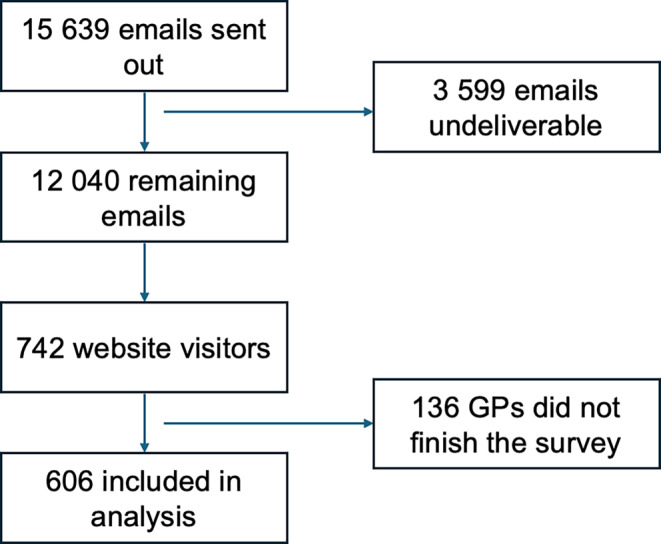
Flowchart

Out of 15,639 emails sent for the survey, 3599 were undeliverable. Of the remaining 12,039 GPs that received an email invitation, 742 visited the survey website and 606 (5.0%) completed the survey, as shown in Fig. 1. The completion rate was 81.7%.

### Professional demographics

Participants’ mean age was 58.5 (± 8.3) years. The sample consisted of 351 male GPs (57.9%) and 249 female GPs (41.1%). The majority, *n* = 282 (46.5%), had individual practices (*KV-Sitz*). Closely followed by physicians working in a group practice (*Gemeinschaftspraxis*; *n* = 245, 40.4%). Most respondents, *n* = 365 (60.2%), had over 20 years of professional experience working as a GP. A total of 315 respondents indicated that they possessed additional qualifications. These were chosen as follows: medicinal tumor therapy (*n* = 5, 0.8%), psychotherapy (*n* = 58, 9.6%), naturopathic medicine (*n* = 121, 20.0%), special pain therapy (*n* = 27, 4.5%), and palliative medicine (*n* = 153, 25.3%); 134 (22.1%) GPs did not have any additional qualification. Besides the ones given, another very common additional qualification—cited through the type-in function in the comment section—was emergency medicine (*n* = 68, 21.5%).

### Clinical experiences and perspectives

The main reasons for consultation were often issues stemming from the cancer or its respective treatment (*n* = 369, 60.9%). Nevertheless, 30.9% of GPs reported that cancer patients seek advice about problems experienced independent of their cancer diagnosis, where the cancer is a secondary diagnosis. However, with this patient group, their secondary cancer diagnosis often comes up during the consultation (*n* = 385, 63.5%). In contrast, others reported that the past cancer diagnosis always (*n* = 131, 21.6%) or sometimes (*n* = 64, 10.6%) comes up. Moreover, this survey represented GPs who were treating more than 15 cancer patients (*n* = 292, 48.2%) at the time of the survey as well as GPs who were seeing those patients a couple of times per month (*n* = 292, 48.2%). Besides 126 GPs reporting that they see their patients once a month, there were still 74 (12.2%) and 91 (15.0%) GPs stating that this patient group visits once or even multiple times a week, respectively.

In terms of the patients’ expectations towards the GP, these seemed to be relatively outbalanced, without much prioritization: psycho-oncologic support (*n* = 451, 74.4%) as well as treating the patient without transferring him/her to another specialist (*n* = 410, 67.7%) seem to be the most relevant duties of GPs. This is closely followed by survivorship care (*n* = 316, 52.1%) as well as transferal to another specialist (*n* = 278, 45.9%).

### Decision-making and perceived competences and needs

**Fig. 2:**
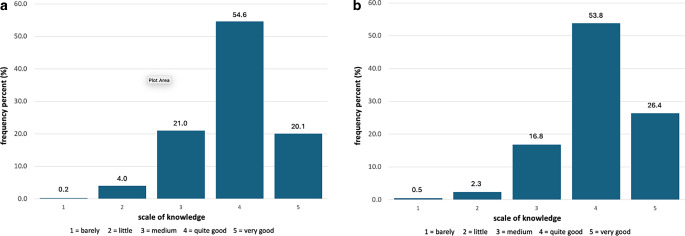
Participants’ knowledge about the symptoms (**a**) and needs (**b**) of cancer patients

A majority of GPs displayed confidence in understanding cancer-related symptoms (54.6%) and addressing patients’ needs (53.8%; Fig. 2a,b, respectively).

**Fig. 3:**
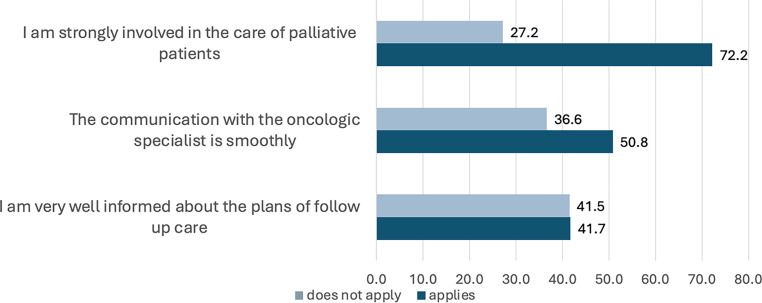
Participants disagreeing/agreeing with statements about their involvement in palliative care

Participants in the study generally reported positive evaluations of the SAPV, with nearly half (48.8%) giving the maximum rating of 5/5 for their services. Additionally, the quality of communication with these networks was rated highly, with 39.6% of participants assigning a positive rating. The data provide a clear view of GPs’ involvement in palliative care, with most respondents (72.3%) expressing a positive assessment of their level of participation. However, communication between GPs and oncologic specialists was reported as an area with varied responses. Furthermore, GPs reported that they are only moderately (*n* = 276, 45.6%) to a little (*n* = 158, 26.1%) involved. Just over one third (36.6%) disagreed that the communication is smooth, while half of the participants (50.8%) agreed. Moreover, knowledge regarding follow-up care plans was evenly split, with 41.5% feeling well informed and an equal proportion not feeling informed enough, highlighting differences in perceived information levels (Fig. 3).

**Fig. 4:**
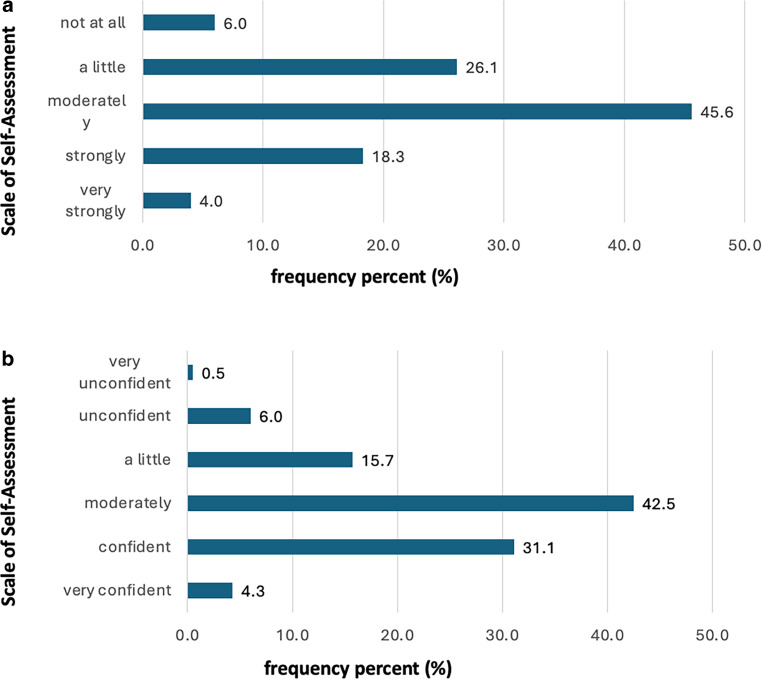
General practitioners’ self-assessment of follow-up care of patients receiving radiation therapy (RT). **a** Degree of involvement, **b** degree of feeling confident in managing RT patients

GPs reported varied levels of involvement (Fig. 4a) and confidence (Fig. 4b) in managing follow-up care for radiation therapy, with 45.5% *moderately* involved and 42.5% *moderately* confident. Notably, a mere 3.9% reported *very strong* engagement in patient aftercare, and only 4.3% expressed a *high degree* of certainty in addressing radiation-induced complications.

A significant majority of the survey participants, comprising 408 individuals or 67.3% of the total respondents, clearly indicated a need for further education in radiation-therapy side effects.

### Interprofessional communication

Respondents were content with the number of oncology patients they were treating (*n* = 544, 89.8%). While 37.8% find the work with cancer patients not burdensome, the majority (*n* = 321, 53.1%) find it a little burdensome, compared to 0.1% who perceive it as very burdensome.

The preferred method of communication among these respondents was via the medical report, with 73.9% (*n* = 448) using this channel and other modalities such as telephone (*n* = 63, 10.4%) and email/fax (*n* = 49, 8.1%) being less commonly employed.

Lastly, GPs perceive their main tasks to be focused on direct medical care on a one-on-one basis (*n* = 276, 45.6%). Besides, other tasks seem to be ranked as having a similar level of importance: coordination and communication with the respective oncologist (*n* = 117, 19.3%), psycho-oncologic care (*n* = 89, 14.7%), sociomedical support (*n* = 67, 11.1%).

### Further comments

A total of 343 respondents used the open-text response option in the final question of the survey. Through thematic analysis, it was evident that several key factors significantly influence the daily professional life of GPs. Besides insufficient staffing and time constraints, these factors encompassed issues related to deficient communication and an undervaluation of their role in the healthcare system.

Firstly, a prominent concern was the communication gap between GPs and specialists, particularly oncologists in university hospitals. GPs reported that they often receive incomplete information or encounter delays in getting medical reports. Sometimes, they would get lab results without sufficient context. Even when medical reports were received, GPs faced the challenge of deciphering the myriad of abbreviations used by various medical specialties.

Secondly, the survey also noted differences in physician–patient interactions across different settings. For example, in MVZs patients are seen by any available doctor, which can be stressful for both patients and doctors. Doctors must quickly familiarize themselves with new patients’ histories, while patients lack a consistent and personal relationship with a single specialist who is thoroughly familiar with their case.

Thirdly, general practitioners feel that their role is often misunderstood or undervalued by their other specialist colleagues. GPs bring to the table a unique long-standing relationship with their patients, fostering a level of trust that is difficult to replicate. GPs have expressed frustration about being left out of ongoing care plans and not being kept informed by other specialists, who tend to take over patient care quite swiftly.

Despite this, the comprehensive care that GPs provide, including ongoing and follow-up care and their psychological and psychosocial support, is undervalued. The established doctor–patient relationship is crucial and often missing in other medical specialties. For instance, during chemotherapy, there is often no consistent point of contact for the patient, a gap which GPs could fill.

GPs also note the necessity of providing accurate and ample information about psychosocial support and alternative therapies, ensuring that patients are legally informed to make shared decisions about their treatment. While palliative care networks are seen as valuable, there is a call for more room to accommodate individual patient wishes within the treatment protocol.

Some GPs offered potential solutions, which were consolidated into the following focal points: introduction of electronic medical reports (e-medical reports), provision of additional training for both GPs and oncologists, and inclusion of family members in the care process.

In addition to electronic records, patients should be provided with a clear and concise follow-up plan and an information sheet about their treatment, copies of which would be included in their medical reports, keeping GPs fully informed. Another suggestion is to facilitate more immediate access to physicians. Implementing coordination checkpoints could solve the issue of managing this information, streamlining the transfer and collection of patient data.

Moreover, the necessity of ongoing education was a significant concern raised. There is a call for comprehensive training programs to address knowledge gaps on both sides: GPs are eager to deepen their understanding of the side effects associated with specific treatment regimens. Furthermore, while GPs are committed to addressing the psychological aspects of cancer care, there is a consensus that oncologists could enhance patient experience by receiving additional training focused on the emotional impacts of cancer treatment. Such educational advancement could be facilitated through mutual training sessions in which GPs and oncologists share insights and expertise.

Lastly, the critical role of involving and transparently communicating with the patient’s family and relatives must not be overlooked. Beyond their decision-making input, relatives can provide invaluable support, as they often know the patient intimately and can more effectively convey the patient’s preferences. This knowledge is crucial for both the GP and the oncologist. Establishing ambulatory counseling centers focused on social medicine could be beneficial in facilitating this aspect of care.

## Discussion

The integration of general practitioners into the oncologic care of patients undergoing radiation therapy remains a largely underexplored area in Germany. Ensuring effective communication and collaboration between GPs and oncologists is crucial for optimizing patient outcomes in cancer care. This study, a cross-sectional online survey conducted across Germany, aimed to investigate the challenges and needs of GPs involved in the care of oncologic patients. Despite a low response rate, the survey produced significant results that require further consideration and discussion.

The primary finding of this study is that GPs are not significantly involved in post-radiation therapy care. This indicates a critical need for improved communication with oncologists and targeted educational initiatives. GPs expressed a strong desire for further education on radiotherapy side effects, emphasizing the necessity of ongoing professional development programs.

GPs have expressed robust confidence in recognizing cancer-related symptoms and addressing patient needs, with more than half rating their knowledge and competencies highly. Nonetheless, this contrasts with the moderate levels of involvement and confidence in managing follow-up care after radiation therapy, where only a small fraction exhibited a very strong engagement or high degree of certainty in managing RT symptoms. The substantial call for further education in radiation oncology, as indicated by two thirds of the respondents, emphasizes a perceived deficiency in current training and underscores the necessity of enhanced educational resources. As novel treatment modalities emerge, it is imperative for GPs to attain a comprehensive understanding of these interventions and their potential side effects. Facilitating access to accredited courses and certifications is essential for equipping GPs to meet these challenges [15, 16].

Consistent with prior studies [17–19], our results reveal a need to redefine the roles of GPs in the care of oncology patients. GPs find themselves at the intersection of coordinating complex care pathways and providing direct medical care.

The investigation by Sandell et al. [20] into the care agreement concordance between GPs and radiation oncologists aligns with our findings, revealing a misperception and underestimation of GPs’ roles and competencies. In our study, only 3.9% of GPs felt very engaged in RT aftercare, and 66% indicated a need for more education in radiation oncology. To bridge this gap, the development of care guidelines and structured clinical assessment checklists is essential. Such tools have the potential to bolster mutual confidence and optimize the care provided by GPs and specialists. Tomascheck et al. [21] reviewed strategies introduced to improve collaboration between GPs and specialists. Common interventions were joint consultations or the discussion of cases, which served the transfer of knowledge. Such interventions were proven to increase satisfaction on both professional sides and improve patients’ health outcomes.

Participants’ satisfaction with SAPV services and their own participation in palliative care suggests effective practices are in place, yet the split in communication quality with oncology specialists and the division in knowledge regarding follow-up care plans reveal critical areas for improvement. The call for additional training is a testament to GPs’ recognition of the evolving complexity of oncologic care and their role within it. According to Peter et al. [22], due to poor knowledge of specialized palliative healthcare, GPs are not being integrated into the medical care of these complex patients, but rather take on coordinating roles with SAPV networks—which they fear might lead to losing track of the patient’s medical history. The complexity of palliative care is further highlighted by Nauck et al. [23], who point out that although SAPV networks are obliged to provide predefined services, specific arrangements such as compensation or service details are negotiated between care providers and the relevant health insurance entities, which can lead to standard contracts applicable to all or necessitate negotiations with regional teams. Thus, depending on various factors such as regional ones, GPs face different challenges in coordinating and communicating the appropriate care. Our survey showed more optimism compared to a Saxony survey on SAPV networks, with younger and female GPs more willing to share care responsibilities. However, there was skepticism about the effectiveness of SAPV. GPs appreciated the idea of consultation and collaborative service provision with SAPV but were reluctant to let SAPV completely take over patient care [24]. While the SAPV concept is deemed very useful and crucial and its services and landscape have expanded since its inception in 2007 [25, 26], challenges in implementation persist. Notably, significant stress for family caregivers and poor communication with other specialists pose barriers to seamless care programs [27]. The current study confirms these issues, finding even more pronounced communication problems between GPs and oncologists or other specialists as compared to communication within SAPV teams.

### Limitations

The study has several limitations. Firstly, the inherent concerns of response rates in survey research are acknowledged. This relatively low response rate is a significant limitation, as it may introduce non-response bias, potentially limiting the generalizability of the findings. It is possible that those who chose to participate may have had differing views or experiences compared to those who did not respond. Despite this shortcoming, the respondents represent a broad demographic of GPs across various practice settings, providing valuable insights into the challenges and needs in oncologic care. Future research should aim to increase response rates by employing additional follow-up reminders and utilizing alternative methods, such as posted letters, to engage a larger proportion of GPs.

While the questionnaire was developed collaboratively by experts in psycho-oncology, radio-oncology, and general medicine and reviewed by experienced physicians, it did not undergo formal validation procedures, such as pilot testing or statistical validation of its psychometric properties due to time constraints. This could have impacted the design and focus. Future studies could benefit from a more formal validation process, including pre-testing with a smaller cohort, to ensure clarity, relevance, and comprehensiveness of the survey items.

Additionally, some email addresses provided by *ArztData AG* were outdated, belonging to retired professionals.

Content-wise, for some participants, this questionnaire might have implied that there is a “one-size-fits-all” approach for cancer patients, which is not the case, as treatments and follow-up care vary significantly. The survey was intended to provide a preliminary understanding of the challenges faced by GPs. Here, it must be highlighted that some tasks may be more relevant than others, and generalizability is difficult.

In conclusion, further research should extend to other medical specialties, to explore the intricate dynamics of the healthcare network.

## Conclusion

GPs in Germany are confident in understanding cancer-related symptoms and patient needs but show moderate involvement in managing post-radiation therapy care. Nearly half rate specialized outpatient palliative care highly, though communication with oncology specialists needs improvement.

A majority of GPs expressed a need for further education in radiation oncology, highlighting a training gap. Addressing this requires initiatives like accredited courses and certifications. Systemic improvements in communication, training, and collaborative care models are essential for better integrating GPs into oncology care.

## Data Availability

The raw data supporting the conclusions of this article will be made available by the authors without undue reservation.
